# Supplementary material for: Vagus nerve stimulation modulates information representation of sustained activity in layer specific manner in the rat auditory cortex
Source: Front Neural Circuits. 2025 Jul 23;19:1569158. doi: 10.3389/fncir.2025.1569158 (PMC12325384; doi:10.3389/fncir.2025.1569158)
Supplement: Supplementary file 1 [file Table_1.DOCX]

Supplementary Material


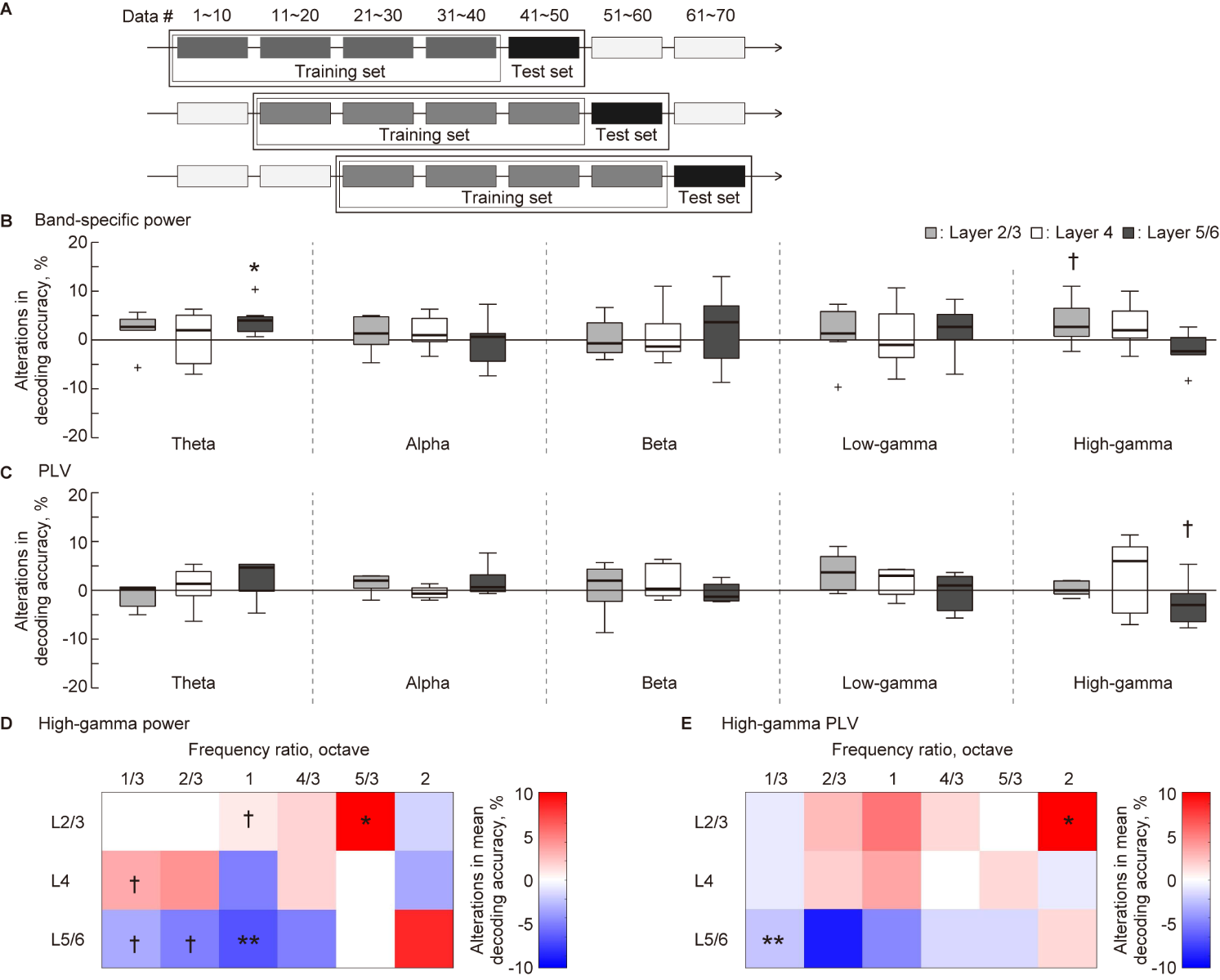


**Supplementary Figure 1.** (A) Considering the effects of the predictability of neural activity occurring within a few seconds, we conducted a similar analysis using the rolling-window method, which is the recommended cross-validation method for time series data. For each test frequency, the 70-input data were divided into groups of 10. Four consecutive groups (40 data) were used for supervised learning, and the subsequent group (10 data) was used to test accuracy. This procedure was repeated three times, resulting in a three-fold cross-validation of the 70-input data. (B and C) As in the main analysis, we obtained the percentage of successful discrimination for the test data (i.e., the accuracy rate) and calculated the mean accuracy rates for all cross-validations and test frequencies for each animal. For the five-choice discrimination of the test frequency, decoding accuracy of pre-VNS activities was subtracted from that of post-VNS activities. We assessed the alterations in decoding accuracy for each frequency band of sustained activity and each layer. Statistical tests were conducted to analyze the changes in decoding accuracy in (B) band-specific power and (C) phase locking value (PLV). Consequently, decoding accuracy significantly increased in L5/6 band-specific power in the theta band, while there was a weak tendency for it to decrease in the PLV in the high-gamma band (Wilcoxon two-sided signed-rank test versus 0%, *p* = 0.016 and 0.094). (D and E) We calculated the difference in mean decoding accuracy between pre- and post-VNS recordings in the two-choice discrimination across six frequency ratios. The red and blue density scales represent the improvement and inhibition of the discrimination, respectively. In L5/6, discrimination between close frequencies, especially those ≤ one octave, tended to be inhibited (*p* = 0.063, 0.052, and 0.0065 for band-specific power and *p* = 0.0050 for PLV). Conversely, in L2/3 and L4, the discrimination accuracy often increased, with some significant increases (*p* = 0.079 and 0.0047 in L2/3, *p* = 0.072 in L4 for band-specific power, and *p* = 0.023 for PLV). Daggers and asterisks indicate a slight or significant increases or decreases in changes in decoding accuracy compared to zero: † *p* < 0.1, * *p* < 0.05, ** *p* < 0.01 (Wilcoxon rank sum test).
